# Chromosome-scale genome assembly of the mangrove climber species *Dalbergia candenatensis*

**DOI:** 10.1038/s41597-024-04032-2

**Published:** 2024-10-31

**Authors:** Miaomiao Shi, Yu Zhang, Huiwen Huang, Shiran Gu, Xiangping Wang, Shijin Li, Zhongtao Zhao, Tieyao Tu

**Affiliations:** 1 State Key Laboratory of Plant Diversity and Specialty Crops/Guangdong Provincial Key Laboratory of Applied Botany/Key Laboratory of National Forestry and Grassland Administration on Plant Conservation and Utilization in Southern China, South China Botanical Garden, Chinese Academy of Sciences, Guangzhou, 510650 China; 2 South China National Botanical Garden, Guangzhou, 510650 China

**Keywords:** Genome, Plant stress responses

## Abstract

Consisting of trees, climbers and herbs exclusively in the intertidal environments, mangrove forest is one of the most extreme and vulnerable ecosystems of our planet and has long been of great interest for biologists and ecologists. Here, we first assembled the chromosome-scale genome of a climber mangrove plant, *D*albergia *candenatensis*. The assembled genome size is approximately 474.55 Mb, with a scaffold N50 of 48.1 Mb, a complete BUSCO score of 98.4%, and a high LTR Assembly Index value of 21. The genome contained 283.46 Mb (59.74%) repetitive sequences, and 29,554 protein-coding genes were predicted, of which 87.54% were functionally annotated in five databases. The high-quality genome assembly and annotation presented herein provide a valuable genomic resource that will expedite genomic and evolutionary studies of mangrove plants and facilitate the elucidation of molecular mechanisms underlying the salt- and water-logging-tolerance of mangrove plants.

## Background & Summary

Mangrove forests, characterized by fluctuating salinity, hypoxia, and intense ultraviolet light in intertidal environments^1^, represent one of the most extreme and vulnerable ecosystems. Despite these challenging conditions, mangroves have evolved a range of distinct morphological and physiological traits in order to adapt the harsh coastal conditions^1–3^, such as vivipary, salt secretion, and aerial roots to adapt. Mangrove forests can mitigate the effects of flooding and typhoons, maintain tropical and subtropical marine biodiversity, and sequester carbon^4^, thereby offering significant ecological benefits and economic value. However, mangrove forests are encountering escalating pressures from global climate changes and anthropogenic activities, such as exploitation and deforestation, which have resulted in more than 20% reduction of area in the past 40 years^4,5^, followed by losses of species richness and functional diversity^6^. This underscores the urgent need for effective conservation, restoration, and management practices to protect the mangrove ecosystems. To achieve successful conservation and sustainable management of mangrove ecosystems, it is essential to gain a deep understanding of the evolutionary patterns and genomic architecture of the diverse flora and fauna species that inhabit these unique habitats. The rapid development of sequencing technologies has enabled numerous studies to successfully generate high-quality whole-genome assembly resources of mangrove plants^2,3,7^, which facilitate uncovering their adaptation mechanisms in the intertidal zone, thereby promoting the breeding of coastal shelterbelts. However, previous studies on mangroves have primarily focused on tree species, with a notable lack of research on shrubs and climbers.

*Dalbergia* L.f. is a genus of the family Leguminosae (Fabaceae), the third-largest plant family of the angiosperms, encompassing approximately 250 species globally of trees, shrubs and lianas^8–10^. These species are predominantly distributed in the pantropic regions of Asia, America, and Africa^11^. Many *Dalbergia* species are economically significant due to the superior quality of their heartwood, characterized by exceptional durability, captivating color, and unique fragrance^12^, such as rosewoods *D. oliveri* Gamble ex Prain, *D. cochinchinensis* Pierre, and *D. odorifera* T. C. Chen, which is widely recognized as “Hongmu” in China^10^. Additionally, some *Dalbergia* species are of ecological importance for their abilities of fixing atmospheric nitrogen with aeschynomenoid type root modules^13^, and functions of ecological restoration in vulnerable ecosystems^14^. High-quality genomic resources provide opportunities to investigate the functional genes associated with key traits and disease resistance, as well as to elucidate the molecular mechanisms underlying environmental adaptation^12,15^. To date, useful genomic data of five species within the *Dalbergia* genus have been recently published, including *D. cochinchinensis*, *D. cultrata*^12^, *D. odorifera*^16^, *D. oliveri*^15^, and *D. sissoo*^17^, all of which are of economic significance due to their valuable heartwood.

**Fig. 1:**
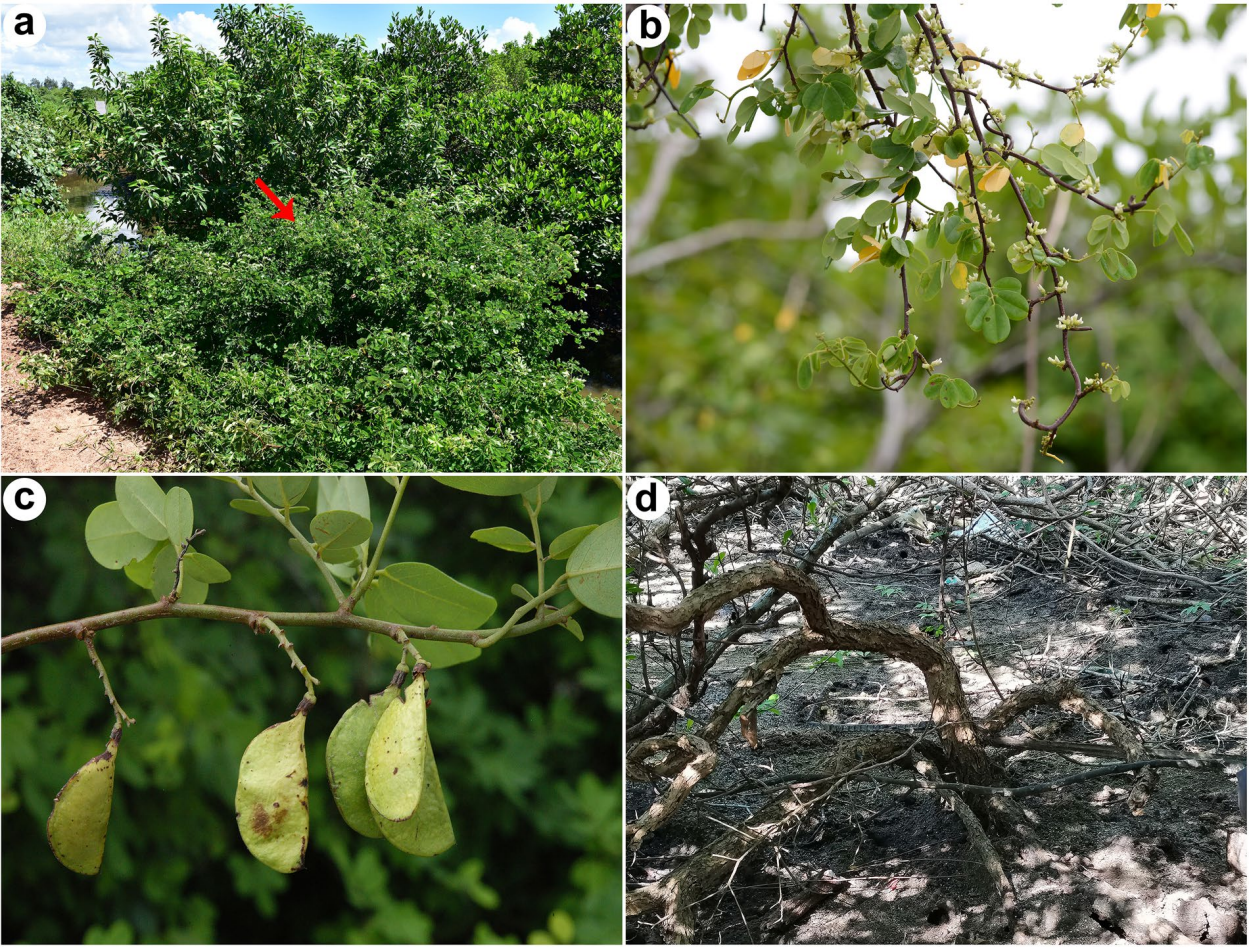
The habitat and morphology of *Dalbergia candenatensis*. (**a**) The intertidal habitat. The red arrow shows *D. candenatensis*. (**b**) Branches bearing flowers. (**c**) Fruits of *D. candenatensis*. (**d**) Woody stem of *D. candenatensis*.

*Dalbergia candenatensis* (Dennst.) Prain (2n = 2x = 20) is a woody climber predominantly found in the tropical coasts of China and neighboring southeast Asian countries, extending south to northern Australia^9^. It is the only species of the genus *Dalbergia* that grows exclusively on the landward side of mangrove forests near the high tide line, categorizing it as semi-mangroves^14^. This species can withstand tidal saline soil and survive under submerged conditions (Fig. 1). As a wood climber, *D. candenatensis* typically climbs on other mangrove plants, and its stem’s high tenacity may enhance the resilience of mangrove forests against wind and waves. The pod’s thick coriaceous or nearly woody nature facilitates long-term fruit floating in seawater and seed dispersal over great distances. Furthermore, the heartwood and leaves of *D. candenatensis* have been reported to contain high concentrations of isoflavonoids, flavonoids, tannins, and phenolic compounds^18–20^, which potentially bolster stress resistance and promote growth in the challenging intertidal zone environments. These morphological and physiological attributes indicate that *D. candenatensis* is well-adapted to its unique habitats, making it as an ideal candidate species for ecological restoration of mangrove ecosystems. However, the lack of the assembled genome has significantly hindered our deeper understanding for *D. candenatensis*’s adaptive mechanisms and its potential application in mangrove forest restoration practices.

Here, we generated a high-quality chromosome-scale genome of a mangrove species *D. candenatensis* (Leguminosae) by combing PacBio high-fidelity (HiFi) long-read sequencing, Illumina short-read sequencing and Hi-C data. The assembled genome had a total size of 474.55 Mb, with a scaffold N50 of 48.1 Mb (Table 1). A total of 471.70 Mb (99.4%) of the sequences were successfully anchored and oriented onto ten pseudo-chromosomes of *D. candenatensis*. The genome contained 29,554 genes and 283.46 Mb (59.74%) repetitive sequences. The high-quality reference genome of *D. candenatensis* provides valuable resource, which will accelerate the genomic and evolutionary studies within the genus *Dalbergia*, facilitate to explore molecular mechanisms involved in the salt- and water-logging-tolerance of mangrove plants, and lay a foundation for utilization in ecological restoration of the mangrove ecosystem.

**Table 1:** Statistics of *Dalbergia candenatensis* genome assembly and annotation.

**Assembly feature**	
Estimated genome size (Mb)	521.35
Assembly size (Mb)	474.55
Scaffold N50 (Mb)	48.1
Contig N50 (Mb)	44.1
Anchor ratio (%)	99.4%
GC content	35.37%
BUSCO (%)	98.4%
LAI	21
**Genome annotation**	
Number of protein-coding genes	29554
Average gene length (bp)	3331.22
Average CDS length (bp)	1127.55
Average exon length (bp)	225.53
**Functional annotation**	
Nr	25831 (87.40%)
eggNOG	24943 (84.40%)
KEGG	10647 (36.03%)
GO	20739 (79.78%)
uniprot	25116 (84.98%)
Total	25871 (87.54%)

## Methods

### Plant materials and sequencing

In June 2021, young and healthy leaves were collected from one individual of *D. candenatensis* for Illumina sequencing, PacBio SMRT sequencing and Hi-C sequencing in Bamen Bay, Hainan, China (110°47′48.43″ E, 19°36′15.28″ N). The voucher specimen was deposited in South China Botanical Garden (accession number: Zsc545). The Cetyltrimethylammonium bromide (CTAB) method was used for genomic DNA extraction^21^. The quality of the genomic DNA was assessed by a NanoDrop spectrophotometer (Thermo Fisher Scientific, USA), using a pure DNA standard with an OD260/280 ratio between 1.8 and 2.0 and an OD260/230 ratio between 2.0 and 2.2. DNA quantification was then performed using a Qubit 4.0 fluorometer (Invitrogen, USA). For Illumina sequencing, libraries with an insert size of 350 bp were prepared for Paired-end sequencing on the Illumina NovaSeq6000 platform. Approximate 34.28 Gb of short-read data was obtained and used for genome survey (Table 2).

**Table 2:** DNA sequencing statistics.

Read_type	Raw data		
	Read_base	Read_Number	Depth (×)
HiFi reads	39,772,719,434	2,444,282	83.91
Illumina reads	34,277,051,700	228,513,678	72.23
Hi-C reads	71,176,276,500	474,508,510	149.99

For PacBio SMRT sequencing, qualified high-quality DNA samples with the bands larger than 30 kb were randomly broken into 15–18 kb fragments, and the libraries obtained by enrichment and purification of large fragments were sequenced on the PacBio Sequel II/PacBio Sequel IIe platform. A total of 39.77 Gb HiFi reads (~83.91 × coverage) with N50 size 17,211 bp were obtained for *de novo* assembling.

The Hi-C library was constructed according to the protocol involving the following steps: fixation of cells using paraformaldehyde to preserve their conformation; cross-linking of DNA in lysate-fixed cells; generation of sticky ends by treating the cross-linked DNA with restriction enzymes; repair and labeling of DNA ends with biotin; connection of DNA fragments using DNA ligase; elimination of cross-linking state and purification of DNA through protease digestion, followed by random fragmentation into 300–500 bp fragments. Subsequently, the library sequencing was performed using Illumina PE150, generating 71.18 Gb reads (~149.99 × coverage). Clean reads were obtained by de-splicing the original sequence and filtering out low-quality reads.

To aid gene prediction, three tissues including leaves, stems, roots of *D. candenatensis* were collected. The total RNA was extracted with HiPure Universal RNA Mini Kit (Magen, Guangzhou, China). Libraries were prepared and sequenced on Illumina NovaSeq6000 platform. A total of 8.25, 6.35 and 6.77 Gb of raw data were generated for leaves, stem, and root samples of *D. candenatensis*, respectively (Table 3).

**Table 3:** RNA sequencing statistics.

Sample	Sequencing platform	Raw data		Clean data	
		Total number of reads (bp)	Total number of bases (bp)	Total number of reads(bp)	Total number of bases(bp)
RNA leaf	Illumina	27,502,891	8,250,867,300	27,011,558	8,103,467,400
RNA stem	Illumina	21,168,213	6,350,463,900	20,545,630	6,163,689,000
RNA root	Illumina	22,555,024	6,766,507,200	21,943,263	6,582,978,900

### Genome survey

**Fig. 2:**
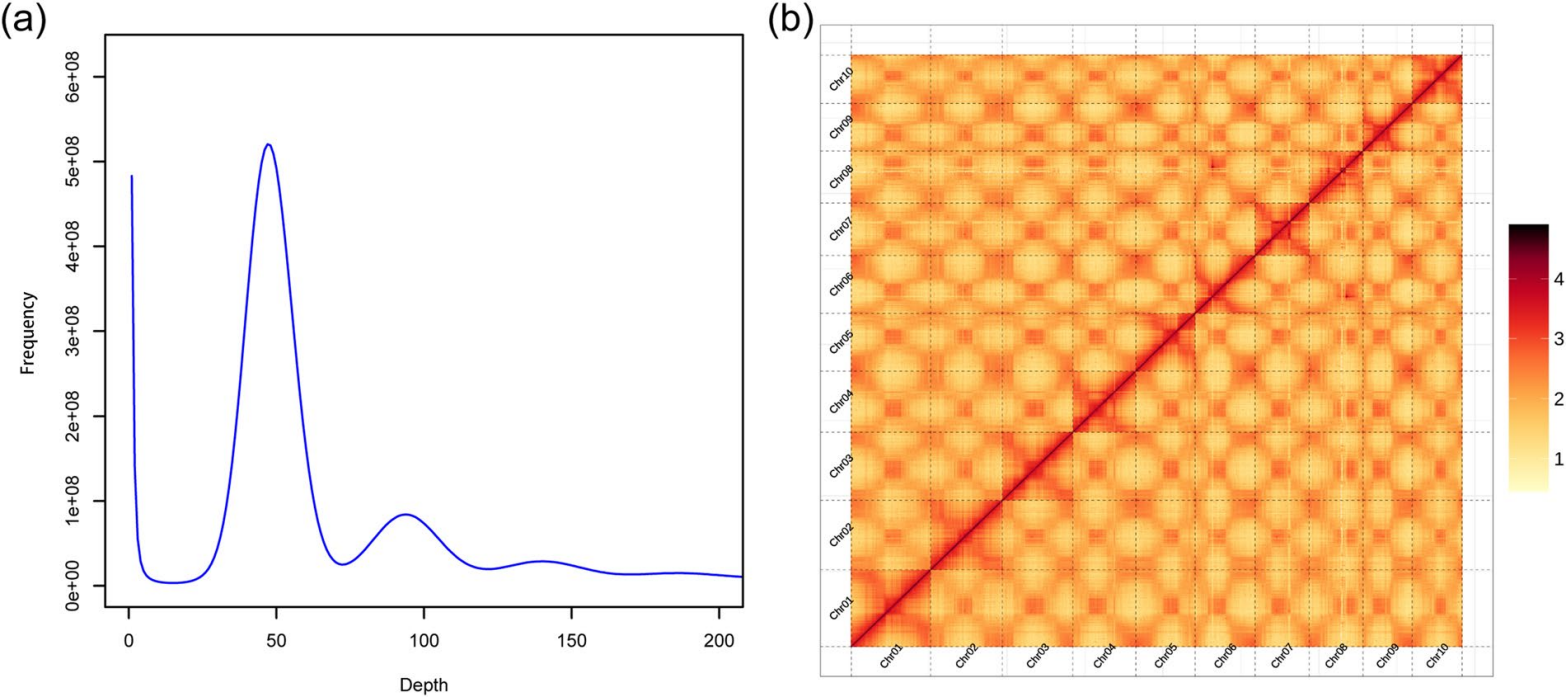
K-mer frequency distribution curve (**a**) and the genome-wide interaction heathap of the *Dalbergia candenatensis* genome based on Hi-C data (**b**).

The Illumina short reads were filtered for the adapter, duplicated and low-quality reads using fastp v0.20.0^22^ with default parameters. To estimate the genome size, heterozygosity and repeat content of *D. candenatensis*, k-mer analysis was performed. The 17-bp k-mers with quality-filtered Illumina short reads were counted using Jellyfish v2.2.7^23^ (Fig. 2a). Based on the counts of k-mers, the genome size of *D. candenatensis* was estimated to be ~521.35 Mb, with a heterozygosity of 0.09% and repeat content of 51.56% using GenomeScope v.2.0^24^.

### *De novo* genome assembly

The PacBio HiFi reads were *de novo* assembled by using HiFiasm v0.16.1-r375^25^ with default parameters. The short DNA reads were aligned to the draft assembled genome by BWA v0.7.17^26^. Subsequently sambamba v1.0^27^ marked the repetitive sequences, and high-quality reads were filtered by samtools. Then polishing the genome assembly was conducted using Pilon^28^ with the parameter (-fix all) for two rounds. To further improve the quality and integrity of the genome, based on the Hi-C data obtained by sequencing, the assembled contigs were scaffolded to the near-chromosome level using AllHiC algorithm^29^, then manually corrected according to the strength of chromosome interactions using juicebox v2.13.07^30^ software. Finally, a genome at the chromosome level was obtained.

The total length of the *D. candenatensis* genome assembly was 474.55 Mb, which is smaller than genome size estimated by k-mer analysis (Table 1). The contig and scaffold N50 values of the genome assembly were 44.1 and 48.1 Mb, respectively. A total of 471.70 Mb (99.4%) of the sequences were successfully anchored to the ten distinct chromosomes (Table 4). The Hi-C interaction map exhibited a pronounced intrachromosomal interaction signal along the diagonal line (Fig. 2b).

**Table 4:** Summary of the ten pseudochromosomes.

ID	No. of contigs	Length (bp)	GC content (%)
Chr01	1	64026954	34.86
Chr02	2	57993263	34.71
Chr03	1	57167204	35.27
Chr04	1	51200502	35.15
Chr05	2	48107494	35.19
Chr06	1	48084155	35.74
Chr07	1	44149702	35.64
Chr08	2	43323290	36.03
Chr09	1	40293102	35.73
Chr10	1	40220275	34.95
unplaced	39	2983011	51.46

### Identification of repetitive elements

High proportion of repetitive sequences in the genome will have a great impact on the accuracy of genome prediction. Therefore, it is necessary to screen the repetitive sequences before gene structure prediction. RepeatModeler v2.0.3^31^ was performed first to identify repetitive sequences based on a *de novo* prediction method, which are as a custom library for annotating repeats using RepeatMasker. Then non-redundant repeats were extracted from Repbase^32^ and Dfam^33^ databases and added to the custom library. RepeatMasker v4.1.2^34^ was used to make predictions for repetitive sequences based on homology searches. DeepTE pipelines^35^ were employed to classify the repeated sequences. A total of approximately 283.46 Mb of the *D. candenatensis* genome was identified as repetitive elements, accounting for 59.74% of the total genome size, among which 275.33 Mb (58.02% of the genome) were annotated as transposable elements (TEs), with LTR (33.14%) being the most abundant TE superfamilies (Table 5).

**Table 5:** Summary of the repetitive sequences in *Dalbergia candenatensis* genome assembly.

Repeat type	Number of elements	Length (bp)	Percentage of sequence
Retrotransposons	283979	168837505	33.93%
SINEs	2709	373980	0.08%
LINEs	25780	3582728	0.72%
CRE/SLACS	41	1752	0.00%
L2/CR1/Rex	3657	141421	0.03%
R1/LOA/Jockey	1789	250739	0.05%
R2/R4/NeSL	883	36528	0.01%
RTE/Bov-B	6303	1204789	0.24%
L1/CIN4	8505	1914749	0.38%
LTR elements	255490	164880797	33.14%
BEL/Pao	1336	76910	0.02%
Ty1/Copia	77181	32608319	6.55%
Gypsy/DIRS1	157524	127678771	25.66%
Retroviral	9956	389277	0.08%
DNA transpsons	374095	78110883	15.70%
hobo-Activator	130231	30335359	6.10%
Tc1-IS630-Pogo	57647	11834351	2.38%
En-Spm	35736	11288382	2.27%
MULE-MuDR	25539	6257480	1.26%
PiggyBac	344	16178	0.00%
Tourist/Harbinger	58767	8976768	1.80%
Other	1173	44235	0.01%
Rolling-circles	9547	1821628	0.37%
unclassified	167619	41720191	8.39%
Total interspersed repeats		288668579	58.02%
Small RNA	9	568	0.00%
Satellites	6946	839701	0.17%
Simple repeats	101763	5946573	1.20%
Low complexity	33510	1721665	0.35%

### Gene prediction and functional annotation

The protein-coding genes in repeat-masked genome of *D. candenatensis* were identified by a combination of methods including ab initio, homologue-based and RNA-seq-based predictions. For ab initio predictions, we employed Augustus v3.4.0^36^, GeneID v1.4^37^, Snap v2006-07-28^38^, GlimmerHMM v3.0.4^39^, GeneMark-ES v4.71_lic^40^ to predict de novo gene models. For homologue-based predictions, GeMoMa v1.9^41^ was used to align the homologous genes from *Arabidopsis thaliana*^42^, *Oryza sativa*^43^ and *D. odorifera*^16^. In addition, adapters, duplicates, and low-quality reads from the RNA sequences of leaves, stems, and roots were filtered using fastp with default parameters, followed by assembly with Trinity v2.13.2^44^, and then PASA v2.4.0^45^ was performed to predict gene model for RNA-seq-based prediction. Subsequently, the results above the three methods were integrated by EVidenceModeler (EVM) v2.1.0^46^ to generate a final non-redundant gene model set. Finally, a total of 29,554 protein-coding genes were identified from repeat-masked genome of *D. candenatensis* (Table 6). The average lengths of genes, coding sequences and exon sequences were 3,331.22 bp, 1,127.55 bp and 225.53 bp, respectively.

**Table 6:** Summary of predicted protein-coding genes in *Dalbergia candenatensis* genome assembly.

Methods	Gene set	Gene number	Average length of gene (bp)	Average number of exon	Average length of CDS (bp)	Average length of exon (bp)	Average length of intron (bp)
**Ab initio annotation**	Augustus	35556	3138.22	4.75	1037.06	218.34	558.80
	GeneID	163926	183.00	1.00	183.00	183.00	360.21
	SNAP	34384	1163.89	2.83	598.75	211.63	421.52
	GlimmerHMM	29606	1989.91	3.53	804.04	227.72	413.82
	GeneMark-ES	28740	4085.25	5.66	1141.64	201.87	408.39
**Homologous annotation**	*Arabidopsis thaliana*	21312	4001.91	5.82	1298.78	223.03	560.08
	*Oryza sativa*	19162	4148.68	6.01	1330.40	221.21	429.28
	*Dalbergia odorifera*	23052	3581.94	5.17	1231.38	238.30	560.32
**Transcriptome annotation**	PASA	179262	3092.54	1.78	757.27	424.50	614.52
**EVM**		29554	3331.22	5.00	1127.55	225.53	557.68

Functional annotation of protein-coding genes was performed by comparing with public databases: non-redundant protein database (NCBI-NR, https://www.ncbi.nlm.nih.gov/), EggNOG^47^ (http://eggnog5.embl.de/), Gene Ontology^48^ (GO, http://geneontology.org/), Kyoto Encyclopedia of Genes and Genomes^49^ (KEGG, https://www.kegg.jp/), Uniprot^50^ (https://www.uniprot.org/), using Diamond v2.0.9.147^51^. In total, 25,871 (87.54%) protein-coding genes of the *D. candenatensis* genome were successfully annotated in functional databases (Table 1).

### Genome-wide synteny analysis

**Fig. 3:**
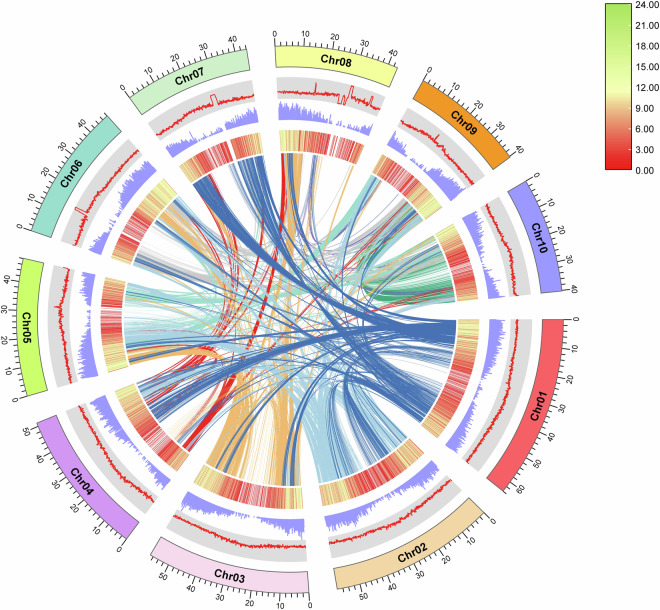
Genomic characteristics of *Dalbergia candenatensis*. The tracks from outer to inner circle represent the ten chromosomes (Chr01-Chr10), GC content, gene position, gene density, and syntenic gene blocks within the genome indicated by connecting lines.

**Fig. 4:**
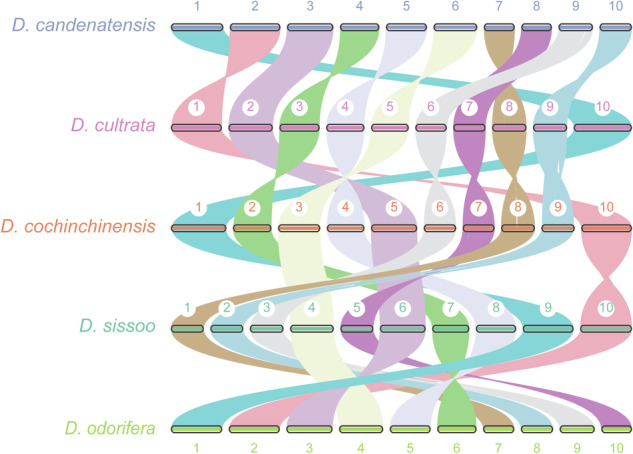
Genome-wide synteny among five genome assemblies in the *Dalbergia* genus. Conserved syntenic blocks were denoted by lines of different colors, each corresponding one of the ten chromosomes.

We identified the syntenic blocks within the *D. candenatensis* genome, as well as between this genome and other published *Dalbergia* genomes, using the python version MCScan implemented in JCVI v1.2.7^52^, with default parameters. Intra-genomic syntenic blocks were visualized using TBtools^53^ (Fig. 3), while inter-genomic syntenic blocks were visualized using JCVI with the parameter -minspan = 30 (Fig. 4).

## Data Records

The raw sequence data have been deposited in the Sequence Read Archive (SRA) at National Center for Biotechnology Information (NCBI) with accession number SRP513077^54^, including PacBio HiFi reads, Illumina PE150 reads, Hi-C reads, and RNA-seq data from different tissues. The final assembled chromosome-scale genome has been deposited in the NCBI GenBank under accession number JBHFQC000000000^55^. In addition, the genome assembly and annotation files were deposited in the Figshare database^56^.

## Technical Validation

By using ~83.91 × PacBio HiFi reads and 149.99 × Hi-C reads, the chromosome-scale genome of *D. candenatensis* was assembled. The assembly was in length of 474.55 Mb with scaffold N50 of 48.1 Mb. The quality of genome assembly was evaluated through following ways. First, inter-genomic syntenic analyses were conducted between *D. candenatensis* and four other *Dalbergia* species to confirm the overall genome structure. Next, to access the integrity of the genome, gene content of the embryophyte odb10 dataset were searched against the assembled genome using Bench-marking Universal Single-copy orthologs (BUSCO) v5.5.0^57^. Additionally, LTR_retriever v2.9.0^58^ was used to calculate LTR Assembly Index (LAI) values using LTR-RTs to assess the assembly continuity. Furthermore, we mapped the PacBio long reads, Illumina short reads, and RNA short reads back to the assembled genome using minimap2 v2.28^59^, BWA v0.7.17^26^, and HISAT2 v2.2.1^60^, respectively, to calculate the mapping rates.

The inter-genomic syntenic analyses revealed high conservation among *D. candenatensis*, *D. cultrata*, *D. cochinchinensis*, *D. sissoo* and *D. odorifera* (Fig. 4), suggesting that the gross genome structure of *D. candenatensis* has been accurately assembled. The complete BUSCO score was 98.4%, of which 95.2% were single-copy genes (Table 7), suggesting a high degree of completeness of the assembly. The LAI value was 21, which reached the “gold standard” (LAI value > 20) of genome assembly proposed by Ou *et al*.^58^. The alignment results showed that 99.92% of PacBio HiFi long reads, 99.76% of Illumina short reads, and an average of 91.6% of RNA reads were successfully mapped to the assembled genome (Table 8). These results indicate a high quality of the genome assembly of *D. candenatensis*.

**Table 7:** BUSCO assessment result.

Type	Number	Percentage
Complete BUSCOs	2290	98.4%
Complete and single-copy BUSCOs	2215	95.2%
Complete and duplicated BUSCOs	75	3.2%
Fragmented BUSCOs	9	0.4%
Missing BUSCOs	27	1.2%
Total BUSCO groups searched	2326

**Table 8:** Statistical summary of mapping rates to the genome assembly.

Read_type	mapping rate(%)
RNA leaf	86.77
RNA stem	97.46
RNA root	90.56
HiFi reads	99.92
illumina reads	99.76
Hi-C reads	99.76

## Data Availability

The software utilized in this study were executed in strict adherence to the official guidelines of published bioinformatics programs. Anything not mentioned in Methods was run with default settings. No custom code was used.
